# Modeling scenarios for mitigating outbreaks in congregate settings

**DOI:** 10.1371/journal.pcbi.1010308

**Published:** 2022-07-20

**Authors:** Seth Blumberg, Phoebe Lu, Ada T. Kwan, Christopher M. Hoover, James O. Lloyd-Smith, David Sears, Stefano M. Bertozzi, Lee Worden

**Affiliations:** 1 University of California San Francisco, Francis I. Proctor Foundation, San Francisco, California, United States of America; 2 Modeling Infectious Diseases in Healthcare Network, Centers for Disease Control and Prevention, Atlanta, Georgia, United States of America; 3 University of California San Francisco, Department of Medicine, San Francisco, California, United States of America; 4 University of California Los Angeles, Department of Ecology and Evolutionary Biology, Los Angeles, California, United States of America; 5 University of California Berkeley, School of Public Health, Berkeley, California, United States of America; 6 University of Washington, Department of Global Health, Seattle, Washington, United States of America; 7 National Institute of Public Health of Mexico, Cuernavaca, Mexico; University of Zurich, SWITZERLAND

## Abstract

The explosive outbreaks of COVID-19 seen in congregate settings such as prisons and nursing homes, has highlighted a critical need for effective outbreak prevention and mitigation strategies for these settings. Here we consider how different types of control interventions impact the expected number of symptomatic infections due to outbreaks. Introduction of disease into the resident population from the community is modeled as a stochastic point process coupled to a branching process, while spread between residents is modeled via a deterministic compartmental model that accounts for depletion of susceptible individuals. Control is modeled as a proportional decrease in the number of susceptible residents, the reproduction number, and/or the proportion of symptomatic infections. This permits a range of assumptions about the density dependence of transmission and modes of protection by vaccination, depopulation and other types of control. We find that vaccination or depopulation can have a greater than linear effect on the expected number of cases. For example, assuming a reproduction number of 3.0 with density-dependent transmission, we find that preemptively reducing the size of the susceptible population by 20% reduced overall disease burden by 47%. In some circumstances, it may be possible to reduce the risk and burden of disease outbreaks by optimizing the way a group of residents are apportioned into distinct residential units. The optimal apportionment may be different depending on whether the goal is to reduce the probability of an outbreak occurring, or the expected number of cases from outbreak dynamics. In other circumstances there may be an opportunity to implement reactive disease control measures in which the number of susceptible individuals is rapidly reduced once an outbreak has been detected to occur. Reactive control is most effective when the reproduction number is not too high, and there is minimal delay in implementing control. We highlight the California state prison system as an example for how these findings provide a quantitative framework for understanding disease transmission in congregate settings. Our approach and accompanying interactive website (https://phoebelu.shinyapps.io/DepopulationModels/) provides a quantitative framework to evaluate the potential impact of policy decisions governing infection control in outbreak settings.

## Author summary

Congregate setting such as prisons and nursing homes pose challenges for infection control. In these settings, vulnerable populations live in dense, highly connected communities. This allows explosive outbreaks of infection such as COVID-19. To reduce the probability of outbreaks occurring and the size of any outbreaks that occur, several control strategies are often available. These include vaccination, depopulation, improving ventilation, and quarantine of affected individuals. In this manuscript, we construct a mathematical model of outbreak dynamics and evaluate the relative effectiveness of different types of control strategies. The model quantifies three phases of outbreak dynamics: the rate of importation of an infection, the probability that an imported infection results in uncontrolled transmission, and the anticipated size of an outbreak caused by uncontrolled transmission. We find that control interventions that decrease both the susceptibility to infection and the transmissibility of affected individuals can have a greater than linear impact on the expected burden of disease. We also find there can be a benefit if residents are apportioned into distinct cohorts. Finally, we examine the relative benefit of preemptive strategies that are enacted prior to an outbreak occurring versus reactive strategies that are deployed once an outbreak has begun.

## Introduction

The COVID-19 pandemic has highlighted the need to quickly identify and implement strategies for controlling the spread of a novel respiratory pathogen. A particular challenge arises in congregate settings such as prisons, nursing homes, and crowded workplaces where transmission is amplified. [1–6] The increased risk of transmission in these settings results in a higher potential for an outbreak to cause many infections within a few weeks. In addition, residents of congregate settings often have a higher prevalence of comorbidities that contribute to worse disease outcomes. [7, 8] The subsequent surge of hospital admissions can strain healthcare capacity and seed increased transmission within the wider community. [9, 10] There are a variety of options to reduce the public health risk associated with congregate settings such as decreasing the number of susceptible individuals via vaccination or depopulation. [10, 11] Control interventions can both decrease the chance of an outbreak occurring and the size of any outbreaks that occur. [12]

To provide a quantitative framework to evaluate the impact of different types of control, we describe a model for the probability of an outbreak occurring in a congregate setting within a specified time period, as well as the size of an outbreak that may occur. We incorporate a model of control that permits a range of assumptions about how control affects outbreak dynamics via density-dependent or frequency-dependent disease transmission. Our model is used to evaluate how control changes the expected burden of disease in congregate settings due to disease outbreaks. We also evaluate the optimal apportionment of residents amongst independent residential units, and characterize when a preemptive control strategy is preferable to a reactive one.

## Methods

### Model overview

**Fig 1:**
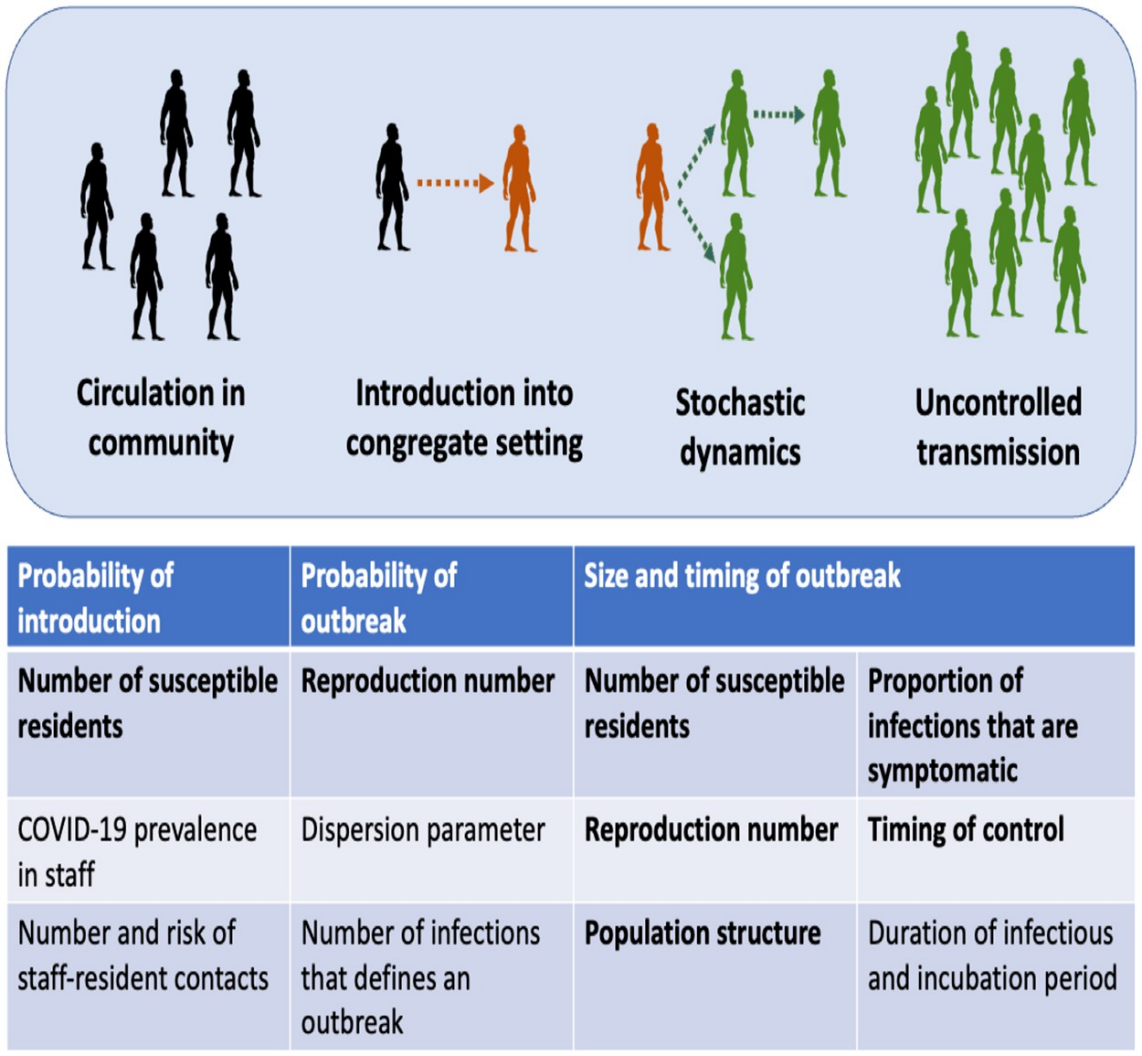
Stages of an outbreak. For an outbreak to occur in a congregate setting, an infection that circulates in the community must be introduced into the congregate setting. Then stochastic dynamics determine whether or not an introduction initiates a large outbreak. Once an outbreak occurs, uncontrolled transmission dictates the size and time course of the outbreak. The table lists the variables that are used in each step of the model. The bolded variables are the variable that are impacted by control interventions that are described in the text.

We structure outbreak dynamics in congregate settings into three stages (Fig 1). First, a case has to be introduced into the congregate setting. This may occur due to direct transfer of an infected case into the congregate population, or from transmission from a staff worker or visitor. We model the primary mode of introduction as being from staff introductions, since this is the hardest type of introduction to repress. Second, an introduction of a case (or a few cases) can either be self-limited or progress to a full outbreak. The probability of an outbreak occurring after an introduction of infection is impacted by the stochastic nature of disease spread, the reproduction number, and the degree of transmission heterogeneity. The reproduction number, *R*, is the average number of infections each new infection causes when all contacts are susceptible to disease. As explained below, we use the negative binomial dispersion parameter to quantify the amount of transmission heterogeneity. Third, if an outbreak is established, the number of symptomatic cases is in proportion to the total number of infected residents. Since the large number of cases overwhelms the stochasticity of transmission, this third stage can be modeled deterministically. We employ a deterministic susceptible-exposed-infectious-recovered (SEIR) compartmental model for this stage. Assumptions of the SEIR model include that the duration of natural or acquired immunity is long enough so that re-infections are unlikely within the time frame of a single outbreak.

We assume that *R* > 1 at the beginning of an outbreak. If *R* were to be less than one, transmission would be self-limited and outbreaks that involve a large portion of the resident population would not be expected. The key outputs for the three stages of the model are the rate of introductions, *ϕ*, the probability that an introduction results in uncontrolled transmission, *P*_*uc*_, and the expected number of symptomatic cases for any outbreak that occurs, *D*_*ob*_. Our models for each stage of an outbreak are described below.

All calculations and simulations are conducted in R, version 4.0.2. Code is available on github (https://github.com/proctor-ucsf/Transmission-in-congregate-settings). An interactive tool for exploring the relationship between input variables and model outputs for each of the three stages of an outbreak is available at https://phoebelu.shinyapps.io/DepopulationModels/.

#### Probability of introduction

We model introduction of disease into the resident community as primarily coming from contact with an infected staff member. The daily rate of introducing a case, *ϕ*, is the product of the average number of staff contacts with susceptible residents per day and the probability that a staff-resident contact causes transmission. The average number of staff contacts with susceptible residents per day is modeled as the product of the number of susceptible individuals in the resident community, *N*_*s*_, and the average number of contacts residents have with staff per day, *N*_*c*_. Note that we define a contact to be a pairing of a resident and staff member. If there are multiple interactions of that pairing through a day, we still count it as one contact. We also ignore staff contacts with residents who are not susceptible as these will not result in introduction of infections. The probability that a staff-resident contact causes transmission is the product of the prevalence of infection in the staff, *P*_*com*_, and the probability that an infectious contact causes an infection *α*_*ic*_,. Thus,

$$\begin{array}{c} \phi =N_{s}\cdot N_{c}\cdot P_{com}\cdot \alpha_{ic}. \end{array}$$ (1)

Our model predicts the average number of days until an introduction occurs is the reciprocal of *ϕ*. If there is significant concern for introduction from visitors, then this can be accommodated by defining *N*_*c*_ as the sum of average daily contacts a resident has with both staff and visitors. The various components of Eq 1 are likely to change over time. For the specific purpose of evaluating the impact that resident-focused control interventions have on the overall disease burden in congregate settings, moderate day-to-day variation in *ϕ* is not expected to have a a significant impact.

#### Probability of an introduction going extinct

When the number of residents with infection is a low number, the stochasticity of transmission can lead to significantly different outcomes. In some cases, an imported infection may lead to no additional infections or a limited number of infections. In other cases, an imported infection may lead to enough cases such that an outbreak ensues. We refer to the probability that an outbreak occurs after an importation as the probability of uncontrolled transmission, *P*_*uc*_.

To model the probability that introduction of disease leads to uncontrolled transmission, we assume that the probability distribution for the number of secondary infections caused by each new infection follows a negative binomial distribution. [13] The negative binomial distribution is described by the reproduction number, *R*, and the dispersion parameter, *k*. The dispersion parameter characterizes the degree of transmission heterogeneity. Low values of *k* are seen when superspreading occurs, meaning that a relatively few number of cases causes a large proportion of onward transmission. We assume that control interventions are implemented in a relatively uniform manner across the resident population so that the value of *k* is not impacted by control efforts.

We define a transmission chain as a group of cases that are connected via transmission events to a single introduction of infection. With the aforementioned assumptions, we can determine the probability, *q*_*s*_, that a single introduction results in a transmission chain having a total of *s* cases before going extinct. As an intermediate variable, we define *r*_*i*→*j*_ to be the probability that *i* infections cause *j* infections in a single generation of transmission. Based on prior work, there is an analytic relationship between *R*, *k*, and *r*_*i*→*j*_, and *q*_*s*_. [14, 15] As previously published,

$$\begin{array}{c} r_{i\to j}=\frac{\Gamma \left(j+k\cdot i\right)}{\Gamma \left(j+1\right)\cdot \Gamma \left(k\cdot i\right)}\cdot {\left(\frac{k}{R+k}\right)}^{k\cdot i}\cdot {\left(\frac{R}{R+k}\right)}^{j}, \end{array}$$ (2)

where Γ is the Gamma function. In addition,

$$\begin{array}{c} q_{s}=\frac{1}{s}\cdot r_{s\to \left(s-1\right)}. \end{array}$$ (3)

The use of a stationary negative binomial offspring distribution assumes that depletion of susceptibles is not a significant factor in transmission dynamics. This is a reasonable assumption if the number of infections that follow an introduction is small due to self-limited transmission. However, a different transmission model is needed that accounts for depletion of susceptibles when an introduction results in uncontrolled transmission. We classify self-limited transmission as occurring every time an introduction leads to a transmission chain that has at most *C*_*th*_ cases. We classify those instances in which an introduction leads to more than *C*_*th*_ cases as uncontrolled transmission. Thus the probability of single introduction leading to uncontrolled transmission is,

$$\begin{array}{c} P_{uc}=1-\sum_{s=1}^{C_{th}}q_{s}. \end{array}$$ (4)

#### Size and temporal dynamics of an outbreak

Once an outbreak is established, we use a deterministic compartmental model to describe the transmission dynamics. In this model, the rate that the susceptible residents become infected is proportional to the product of the number of susceptible and infectious residents. Once infected, a resident will be classified as exposed, but not infectious. Exposed residents transition into being infectious at a constant per capita rate. Infectious residents are removed from the population at a constant per capita rate. Removal may occur due to recovery, transport to a higher level of care or death. Once residents are removed, they are considered non-infectious and are no longer susceptible. During the course of an outbreak, it is assumed that a negligible number of new susceptible or infectious residents are introduced into the population. The equations describing the model are,

$$\frac{dS}{dt}=-\frac{S}{N}\cdot \frac{R\cdot I}{T_{I}}$$ (5)

$$\frac{dE}{dt}=\frac{S}{N}\cdot \frac{R\cdot I}{T_{I}}-\frac{E}{T_{E}}$$ (6)

$$\frac{dI}{dt}=\frac{E}{T_{E}}-\frac{I}{T_{I}}$$ (7)

$$\frac{dX}{dt}=\frac{I}{T_{I}}$$ (8)

where *S*, *E*, *I*, and *X* represent the number of susceptible, exposed, infectious, and removed residents. The reproduction number prior to depletion of susceptibles from uncontrolled transmission, is denoted by *R*. The recovered compartment is labeled *X* so as not to confuse it with *R*_*B*_. The average time for being in the exposed and infectious states is represented by *T*_*E*_ and *T*_*I*_, respectively. As explained below, the way in which *R* is modelled to change with the degree of control will dictate how control interventions act in a frequency-dependent versus density-dependent manner. [16] However, once uncontrolled transmission occurs, we assume outbreaks behave in a density-dependent manner, meaning that the hazard for infection is directly proportional to the number of infected residents.

To determine the total number of residents infected, *I*_*ob*_, the total number of residents with symptomatic disease, *D*_*ob*_, the maximum number of residents infected at once, *M*_*T*_, and the time of peak incidence, *T*_*M*_, we numerically compute the time series for our deterministic SEIR model using time steps of 0.2 days. Then,

$$I_{ob}=X\left(t\to \infty \right)$$ (9)

$$D_{ob}=\rho \cdot I_{ob}$$ (10)

$$M_{T}=\underset{t\in \left(0,\infty \right)}{\text{max}}\;I\left(t\right)$$ (11)

$$T_{M}=t\;\;\text{when}\;\;I\left(t\right)=M_{T},$$ (12)

where *ρ* is the proportion of infected residents who develop symptomatic disease. We use ‘case’ to designate a resident with symptomatic disease.

For the simple compartmental model we are utilizing, other methods are available for determining the attack rate, such as transcendental equation relationships for the final size of epidemics. [17] The benefit of utilizing a simulation approach is that it offers a more flexible framework for making modifications to the model such as for different risk classes, more complex population structure, or time-varying parameters (e.g. reactive control scenario presented below).

### Impact of preemptive control

Each of the stages of our model for outbreak dynamics is influenced by the number of susceptible individuals, *R*, the proportion of infections that are symptomatic, or a mix of these three quantities. The way that a control intervention changes each of these quantities serves as the basis for assessing how control impacts the burden of disease in congregate settings.

We define *N*_*B*_, *R*_*B*_, and *ρ*_*B*_ as the baseline values for *N*, *R*, and *ρ* prior to any control interventions, and prior to any outbreak occurring. We let *γ* represent the ‘Degree of control’ that is contributed by a particular intervention. Control is modeled to have three possible effects, parameterized by three different control indicies: Θ_*N*_, Θ_*R*_, and Θ_*ρ*_. The degree to which control impact the size of the susceptible population, the reproduction number and the proportion of symptomatic infections is modeled as,

$$N=\left(1-\Theta_{N}\gamma \right)N_{B}.$$ (13)

$$R=\left(1-\Theta_{R}\gamma \right)R_{B}.$$ (14)

$$\rho =\left(1-\Theta_{\rho }\gamma \right)\rho_{B}.$$ (15)

For each of *N*, *R*, and *ρ*, decrease occurs linearly with respect to the control parameter, *γ*. Each of Θ_*N*_, Θ_*R*_ and Θ_*ρ*_ vary between zero and one. Values of zero for each of the Θ parameters indicate the associated variable is not impacted by control. Values of one indicate that the associated variable decreases in direct proportion to *γ*. We assume that the dispersion parameter is independent of *γ*.

Different types of control interventions correspond to different combinations of Θ_*N*_, Θ_*R*_ and Θ_*ρ*_ (Table 1). For depopulation, Θ_*N*_ is always one since control directly removes susceptible individuals from the population. For Θ_*R*_ = 0, the reproduction number remains constant even when control is applied, while Θ_*R*_ = 1 means that perfect control will results in an *R* of zero. In disease dynamics literature Θ_*R*_ = 0 and Θ_*R*_ = 1 may be referred to as frequency-dependent and density-dependent transmission respectively. [16] Thus, for depopulation Θ_*R*_ = 0 is consistent with the mean number of contacts per resident remaining constant even when the population size is reduced. Meanwhile, Θ_*R*_ = 1 corresponds to resident contacts being reduced in proportion to the population size. In the case of vaccination, Θ_*R*_ = 0 is equivalent to a vaccine that allows people to catch and spread the virus, while remaining asymptomatic. This is distinct from frequency-dependent depopulation because asymptomatic cases can still serve as an index case of an outbreak (i.e., Θ_*ρ*_ = 1). Meanwhile, vaccination with Θ_*R*_ = 1 corresponds to a vaccine that is equally effective at reducing disease and transmission. Thus density-dependent depopulation and density-dependent vaccination are modeled the same way. Intermediate values of Θ_*R*_ may occur in many ways. Examples include poor ventilation systems in which halving the number of infected neighbors does not half the risk of acquiring disease, or vaccines that provide only partial protection against infection even when protecting against disease. Our modeling framework also allows investigation of interventions that decrease *R* without changing the size of the susceptible population or proportion of asymptomatic infection (Θ_*R*_ = 1 and Θ_*N*_ = Θ_*ρ*_ = 0). This might be due to measures that improve ventilation, social distancing, or use of personal protective equipment.

**Table 1:** Different types of outbreak mitigation efforts can be modeled.

Mechanism of control	Θ_*N*_	Θ_*R*_	Θ_*ρ*_
Depopulation (frequency-dependent)	1	0	0
Vaccination (frequency-dependent)	0	0	1
Depopulation or vaccination (density-dependent)	1	1	0
Transmission reduction only	0	1	0

A key difference between depopulation and vaccination is that for frequency-dependent transmission (i.e., Θ_*R*_ = 0), depopulation reduces the number of susceptible individuals (i.e., Θ_*N*_ = 1) while vaccination does not. Instead vaccination simply decreases the proportion of infections that are symptomatic (i.e., Θ_*ρ*_ = 1). Thus, in the density-dependent scenario, depopulation impacts both the probability of an imported infection and the burden of disease caused by an outbreak (S5 Fig). However, vaccination would not change the probability of an outbreak occurring, but would decrease the burden of disease.

To model the overall impact of depopulation, we define *D*_*overall*_ to be the average number of symptomatic cases expected due to outbreaks that are initiated over a defined time interval, *T*. The expected number of introductions is *ϕT*. The overall probability, *P*_*ob*_ of an outbreak occurring is one minus the probability that no introductions lead to uncontrolled transmission. The average number of cases expected due to outbreak dynamics is the overall probability that an outbreak occurs times the expected number of symptomatic cases per outbreak. That is,

$$P_{ob}=1-{\left(1-P_{uc}\right)}^{\phi T}$$ (16)

$$D_{overall}=P_{ob}\cdot D_{ob}$$ (17)

Here we have assumed that only one uncontrolled outbreak can occur in a residential unit. For our SEIR model the effective reproduction number will be less than one after an outbreak occurs, and so this assumption is equivalent to population turnover being slow to replenish the susceptible population. This is a reasonable assumption in congregate facilities such as assisted living facilities and prisons, where the average length of stay is high compared to the duration of an outbreak. By having a fixed value for *ϕ* rather than a distribution of values, we have also assumed that the rate of a stochastic point process governing introduction of infections is constant with time. This ignores the potential clustering of imported infections from the community. The overall impact of control is probed by evaluating how *D*_*overall*_ depends on *γ*.

In the next two subsections, we extend our model to consider two decision-making scenarios. First, we consider how the average number of cases from outbreaks depends on how a fixed number of residents are apportioned into two independent residential units. Second, in contrast to the preemptive strategy of implementing control before an outbreak occurs, we consider how the average number of cases from outbreak dynamics is impacted by a reactive control strategy in which control is implemented only after uncontrolled transmission is detected.

#### Optimizing distribution of residents

When considering the optimal proportion of residents to house in each of two independent residential units, we specify the baseline occupancy of each residential unit, $N_{B}^{1}$ and $N_{B}^{2}$, as well as the basic reproduction numbers at those baseline occupancies assuming all residents are susceptible, $R_{B}^{1}$ and $R_{B}^{2}$. We use *γ* to specify the degree of control that is applied to the combined population. To explore the scenario anticipated to have the greatest impact on how residents are apportioned, we assume density-dependent control (i.e. Θ_*N*_ = Θ_*R*_ = 1, Θ_*ρ*_ = 0). We allow the proportion, *σ*, of residents assigned to residential unit one to be adjusted between zero and one. We define $N_{adj}^{1}$ and $N_{adj}^{2}$ to be the adjusted sizes of the residential populations. We define $R_{adj}^{1}$ and $R_{adj}^{2}$ to be the adjusted reproduction numbers of each residential unit prior to any outbreak occurring. Then,

$$N_{adj}^{1}=\sigma \cdot \left(N_{B}^{1}+N_{B}^{2}\right)$$ (18)

$$N_{adj}^{2}=\left(1-\sigma \right)\cdot \left(N_{B}^{1}+N_{B}^{2}\right)$$ (19)

$$R_{adj}^{1}=R_{B}^{1}\cdot \frac{N_{adj}^{1}\cdot \left(1-\gamma \right)}{N_{B}^{1}}$$ (20)

$$R_{adj}^{2}=R_{B}^{2}\cdot \frac{N_{adj}^{2}\cdot \left(1-\gamma \right)}{N_{B}^{2}}$$ (21)

For simplicity and illustrative purposes, this model assumes that the same amount of control is applied to each residential unit and the only variable to adjust is the proportion of residents assigned to each unit. Given these parameterizations, the three stages of our model for outbreak dynamics is run for each residential unit independently. This determines each unit’s outbreak probabilities, $P_{ob}^{1}$ and $P_{ob}^{2}$, and expected outbreak sizes, $D_{ob}^{1}$ and $D_{ob}^{2}$. The joint probability of an outbreak occurring in at least one residential unit, $P_{ob}^{all}$, and the overall expected number of cases due to outbreak dynamics, *D*_*overall*_, is then,

$$P_{ob}^{all}=P_{ob}^{1}+P_{ob}^{2}-P_{ob}^{1}\cdot P_{ob}^{2}$$ (22)

$$D_{overall}=P_{ob}^{1}\cdot D_{ob}^{1}+P_{ob}^{2}\cdot D_{ob}^{2}$$ (23)

#### Reactive control

As an alternative to implementing control before an outbreak occurs, we also consider a reactive control approach. To evaluate the maximal impact that reactive control can have, density-dependent control is assumed (i.e. Θ_*N*_ = Θ_*R*_ = 1, Θ_*ρ*_ = 0). In this scenario, we allow the baseline model to run until a ‘trigger threshold’ is reached for the number of infection and then reactive control is initiated. Once reactive control is initiated, the model continues to run unchanged for a time period specified by the control delay. The control delay might represent a delay in implementing depopulation due to logistical barriers, or the effective delay in vaccine efficacy due to the time it takes to achieve a biological response after vaccination. Although there will be individual level heterogeneity in the control delay, we assume that we can represent the population-level control delay as a single number. After the control delay is completed, the susceptible population experiences a one time relative decrease of one minus the reactive control efficacy, *γ*_*r*_. The reactive control efficacy is distinct from the degree of preemptive control denoted by *γ* used above. The rest of the model proceeds as before.

### Parameter estimates

The value of the reproduction number is of particular importance for determining the relative impact of control interventions. For any one disease, there is often great variability of reproduction number estimates. For SARS-CoV-2, there are published estimates of the reproduction number as high as 8.4 in prison settings. [18] Meanwhile less transmissible diseases can also cause outbreaks in congregate settings, such as tuberculosis in prisons or influenza in nursing homes. [19–23] Given the range of estimates for the reproduction number of any single disease, including variants of concern, we explore a range of values for the baseline reproduction number, *R*_*B*_. For the purpose of our analyses, *R*_*B*_ is includes system-wide control interventions that are in place before consideration of whichever intervention is being specifically analyzed with our model. That is *R*_*B*_ may incorporate interventions such as masking, and social distancing that are not being explicitly modelled here.

To provide context for our analyses, we focus on the spread of SARS-CoV-2 within the California prison system between April 2020 and February 2021 for choosing the remaining parameters. Based on current literature for SARS-CoV-2, we assume an average latent period of three days (i.e. time between the occurrence of infection and the onset of infectiousness), and an average infectious period of seven days [24–28] We assume that 75% of infections produce symptomatic disease. [29] The prevalence of infection in the staff, the average daily number of staff contacts each resident has, and the probability that an infected staff member transmits disease to a resident during a contact are estimated as 0.01%, 10 and 1% respectively based on a combination of community dynamics and the empirical observation of relatively frequent outbreaks occurring in the CDCR institutions. [30] The average population size of the 35 CDCR prisons was about 3,300 at the beginning of 2020. [31] Meanwhile prisons typically consist of multiple buildings, that have a degree of independence. Thus, to approximate a single congregate population, we choose a population size of 1,000 for our analyses. Since our analyses focuses mostly on the relative rather than absolute impact of control interventions, the exact proportion of infections that are symptomatic and the specific size of the modeled populations are of secondary importance.

Numerous studies have shown that respiratory diseases often exhibit superspreading behavior, characterized by a dispersion parameter for the negative binomial offspring distribution that is less than one. [13] Recent studies indicate that SARS-CoV-2 follows this pattern. Thus we assume a value of 0.2 for the dispersion parameter, *k*, utilized in our calculation for the probability that a single introduction of infection will lead to a disease outbreak. [32–34]

## Results

### Case example: Evaluating model assumptions in California State Prisons

To illustrate the applicability of our model, we utilize publicly available data from the California Department of Corrections and Rehabilitation (CDCR) for the spread of SARS-CoV-2 in California State Prisons. [30] From the beginning of CDCR reporting in April 2020 through February 2, 2021 there were over 41,000 SARS-CoV-2 cases in California State prisons. Within each institution, periods of low disease prevalence were interrupted by focal outbreaks caused by rapid spread (S1 Fig). Some institutions have multiple outbreaks, which is consistent with separate residential buildings on a campus having outbreaks at different times. Meanwhile there are many examples of small clusters of cases that do not progress to an outbreak. Upon aggregating case counts by institution and then assigning clusters of infection based on having at least fourteen days of no cases between clusters, we find that 23% of clusters consist of an isolated case and 53% of clusters have greater than ten cases suggestive of a large outbreak. Overall this data supports our key assumptions that outbreak dynamics are initiated by sporadic introductions into the residential community and that stochastic dynamics determine the probability that a sporadic introduction progresses into an uncontrolled outbreak.

The data also exhibit explosive outbreaks where hundreds of cases can occur in the span of a few weeks. Such dynamics are congruent with our approach to modeling the size of an outbreak with a deterministic model that assumes large groups of residents behave as a well mixed population. Aspects of prison life which support the high degree of connectedness among residents include overcrowded conditions in which large numbers of residents reside in a single large dormitory room, and poor ventilation systems that can potentially circulate respiratory pathogens between cells. [35] There are other aspects in which residents may not be well mixed, such as those in high security individual cells. Thus the applicability of model assumptions depend on both the mode of transmission and the specific set or residents considered.

### Stages of outbreak dynamics

A prerequisite for an outbreak to occur is introduction of disease into the residential community. For our model, the frequency of introductions increases with a larger residential population, higher prevalence of infection in the community, higher resident-staff contact rate, or higher probability that a resident contact with an infected staff member transmits a new infection (S2 Fig).

An introduction of infection may or may not lead to an outbreak. The probability of an outbreak increases as the number of introductions, or the reproduction number increases (S3 Fig). This probability also depends on the dispersion parameter. High values of the dispersion parameter correspond to homogeneous transmission and more predictable dynamics, whereas low values correspond to heterogeneous dynamics that are more likely to produce either explosive outbreaks or dead-ends to transmission. Thus high values of the dispersion parameter lead to a higher outbreak probability.

Once an outbreak occurs, the number of infectious individuals in our model grows exponentially until there is a significant depletion of susceptible individuals (S4 Fig). A reproduction number above one is necessary for an outbreak to occur, but even values moderately above one lead to a large attack rate (i.e., proportion of infected residents). A reproduction number as low as 1.5 results in an attack rate of 59%, and a reproduction number of 2.5 leads to an attack rate of 89% (as seen by the asymptotic value for the number of removed individuals in the two panels of S4 Fig).

### Impact of preemptive control

**Fig 2:**
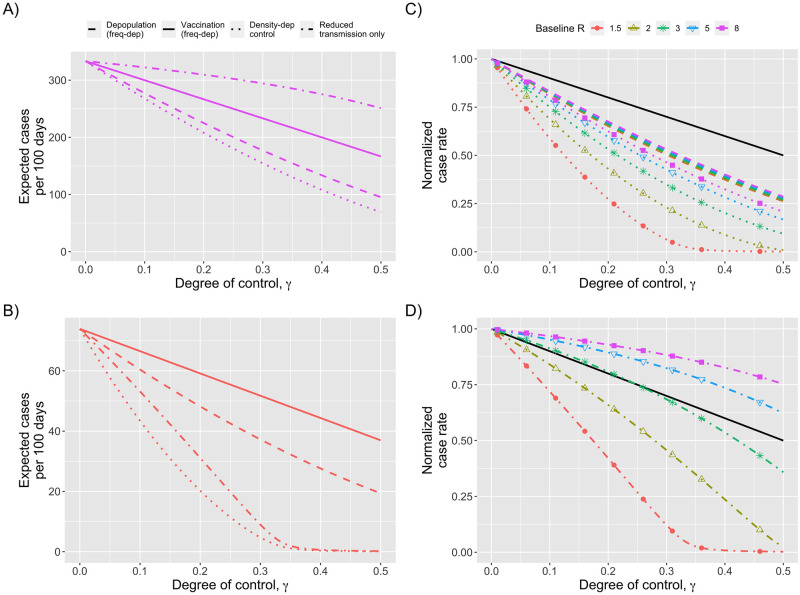
Impact of control measures on decreasing the burden of disease outbreaks in congregate populations. A) The expected number of cases due to outbreaks occurring within 100 days as a function of control. The degree of control, *γ*, shown on the x-axis provides a continuous parameterization from no control (*γ* = 0) to complete eradication of disease (*γ* = 1). Each line represents a different mechanism of control as indicated by the legend and modeled per Table 1. The baseline reproduction number is constant for all modes of control (*R*_*B*_ = 8). B) Analogous to panel A, except that *R*_*B*_ = 1.5. C) Analogous to previous panels, except that the expected number of cases has been normalized to a rate of one when the level of control is zero. Colors and shapes correspond to different values of the baseline reproduction number, *R*_*B*_, as specified by the legend. When vaccination does not change *R* (frequency-dependent), the normalized case rate is independent of *R*_*B*_. This is represented by the single black line. All mechanisms of control shown are variations of vaccination or depopulation. Thus the *γ* value shown on the x-axis can be interpreted as the proportion of the population that has been directly protected from acquiring disease (and possibly infection as well). D) Similar to panel C except that the colored lines now represent a control intervention that effects *R* without changing the number of susceptible individuals. Thus the *γ* value shown on the x-axis is the proportion that the reproduction number has decreased from the baseline value of *R*_*B*_.

When our models of introduction of infection, outbreak probability and outbreak size are combined, there can be a significant impact of control interventions (Fig 2). Given that there is likely substantial variability in the value of the reproduction number within different congregate settings, it is notable that control interventions have a large impact for a wide array of *R*_*B*_ values. When both the susceptible population and reproduction number is decreased by 20% via vaccination or depopulation (i.e. *γ* = 0.2, and Θ_*N*_ = Θ_*R*_ = 1), the expected number of total cases decreases by 73%, 47%, 40%, and 38%, for *R*_*B*_ of 1.5, 3.0, 5.0, and 8.0 respectively.

The mechanism of control changes the overall impact of interventions (Fig 2A and 2B). Interventions that decrease the susceptibility of residents to infection have the most impact (e.g., depopulation or density-dependent vaccination that protects against infection). When the reproduction number is high, the benefit of interventions that only change the ratio of symptomatic infection are greater those that change the reproduction number. However, when the reproduction number is low, any interventions that change the reproduction number are increasingly helpful (in relative terms).

Notably, the reduction in the number of cases as a function of control occurs in greater than linear fashion for depopulation (seen by colored lines falling below the black line in Fig 2C). The decrease is even more significant if control decreases the transmission potential of individuals, as compared to control affecting susceptibility alone (seen by dotted lines falling below the dashed lines in Fig 2C). The greater than linear impact of control can be explained by how the expected number of cases is the product of the probability of an introduction, the probability that an introduction leads to an outbreak and the size of an outbreak. All of these factors depends on either the effective reproduction number, the size of the susceptible population, or both (Fig 1). Thus each stage of outbreak dynamics contributes to the reduction in the number of cases when control is applied.

**Fig 3:**
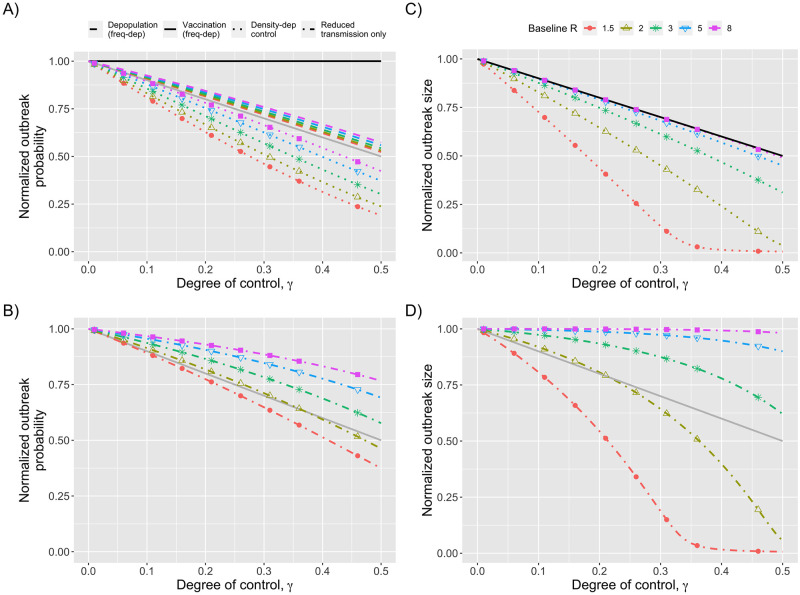
Probability and size of an outbreak. In panels A and B, the probability of an outbreak occurring in 100 days is shown as a function of control. In panels C and D, the expected number of cases per outbreak are shown. All panels show normalized values that are always one when there is no control. Different colors and shapes represent different values of the baseline reproduction number, *R*_*B*_. In panels A and C, the degree of control can be interpreted as the proportion of the susceptible population reduced by processes such as vaccination or depopulation. In panels B and D, the degree of control reflects the proportion that the baseline reproduction number is decreased. In panels A and C, the black line corresponds to frequency-dependent vaccination, which is independent of the baseline reproduction number. In panel C, the black line also corresponds to frequency-dependent depopulation. The grey line in panels A, B, and D is a visual aid to show what results would be expected if the normalized probability or output size varied in direct proportion to the degree of control. A similar grey line is absent in panel C because it overlaps directly with the black line.

Depending on public health priorities, it may be helpful to consider the probability of an outbreak occurring, or the expected size of an outbreak separately from the overall burden of cases expected from outbreak dynamics (Fig 3) For density dependent control the combined effect of a decreased rate of importing an infection and a decreased probability of an importation causing an outbreak means there is a greater than linear decrease in the probability of an outbreak occurring. When control does not affect transmission, there is a less than linear impact on the probability of an outbreak primarily because control does not change the probability that an outbreak occurs after an introduction of infection (non-linearity between the rate of importation of infection and the outbreak probability contributes a smaller effect). For density dependent transmission there is also a greater than linear impact of control on the expected size of an outbreak(Fig 3C). In contrast, when control only impacts the number of individuals susceptible to disease, there is a linear relationship between control and the size of an outbreak.

**Fig 4:**
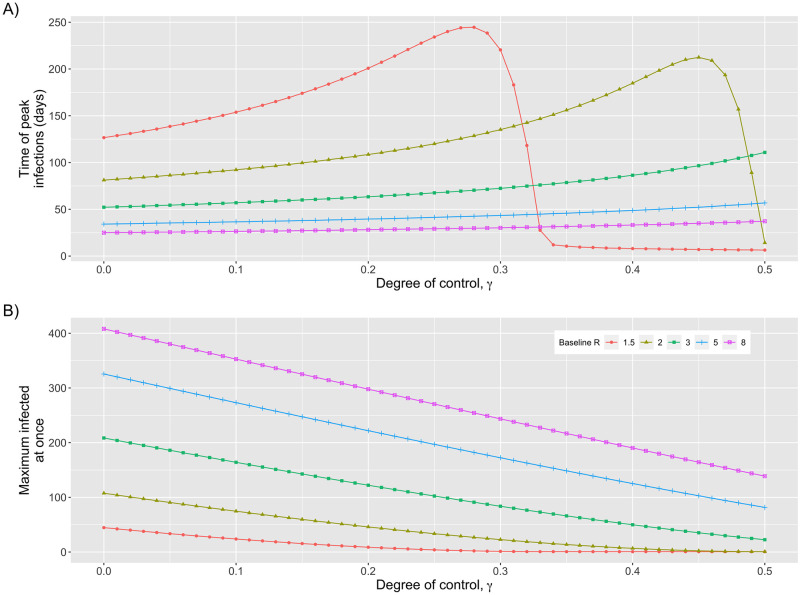
Peak of outbreak. A) Time until peak in the number of infectious individuals, measured from the start of an outbreak B) Maximum number of residents who are infectious at once. For both panels density-dependent control is assumed (i.e. Θ_*N*_ = Θ_*R*_ = 1). Colors and shapes represent different values of the baseline reproduction number, *R*_*B*_, as indicated by the legend. Parameter values are the same as for S4 Fig. For lower values of baseline *R*_*B*_ the timing of the peak in the number of infectious individuals abruptly decreases when the control factor is sufficiently high because the effective reproduction number becomes less than one and thus no significant peak in infectious cases occurs.

Besides a decrease in overall disease, our model predicts a couple other changes in the dynamics of outbreaks as interventions that impact transmission are applied (i.e. Θ_*R*_ = 1). First, reducing the size of the susceptible population also delays the timing of the peak for the number of infectious individuals (Fig 4A). Second, it reduces the maximum number of individuals infected at once (Fig 4B). These factors can help facilitate roll-out of public health interventions that can further decrease the burden of disease. It also helps to minimize overcrowding of healthcare facilities used to treat severe cases. If control has no impact on the reproduction number (i.e. Θ_*R*_ = 0), our model predicts that control has no impact on the temporal dynamics of uncontrolled outbreaks.

When an intervention only impacts the percent of infections that have symptomatic disease (i.e. a vaccination that prevents disease but not transmission), our model predicts a linear relationship between case burden and the degree of control (Fig 2C). That is because when an intervention does not impact transmission, the probability of an outbreak remains constant, and the expected number of cases per outbreak decreases linearly with control (as shown by black line in Fig 3A and 3C).

The efficacy of interventions that focus solely on decreasing transmission without any impact on susceptibility (e.g. improving ventilation or social distancing) have substantial dependence on the baseline reproduction number. When the reproduction number is high, control has less effect than decreasing the proportion of symptomatic cases via frequency-dependent vaccination. This is because disease transmission continues to approach the saturation limit for the probability of an outbreak occurring as well as the proportion of residents who become infected when an outbreak occurs (as shown by how the colored lines for high *R*_*B*_ values remain above grey line in Fig 3B and 3D). However, if the baseline reproduction number is low enough that a moderate level of control can bring the effective reproduction number below the critical value of one, then control of transmission can have a substantially greater impact (as shown by how the *R*_*B*_ = 1.5 line falls below the grey line in Fig 3B and 3D).

#### Optimizing distribution of residents

When heterogeneity in contact patterns is present, the optimal strategy for distributing immunization or other control efforts will depend on the details of the population structure. [36, 37] This raises the possibility that introducing heterogeneity by cohorting residents into two independent residential units may offer a method of outbreak control that complements other control efforts. Independent residential units might be different buildings within a prison, different units in a healthcare facility, or other housing paradigms that exclude mixing of residents from different units.

**Fig 5:**
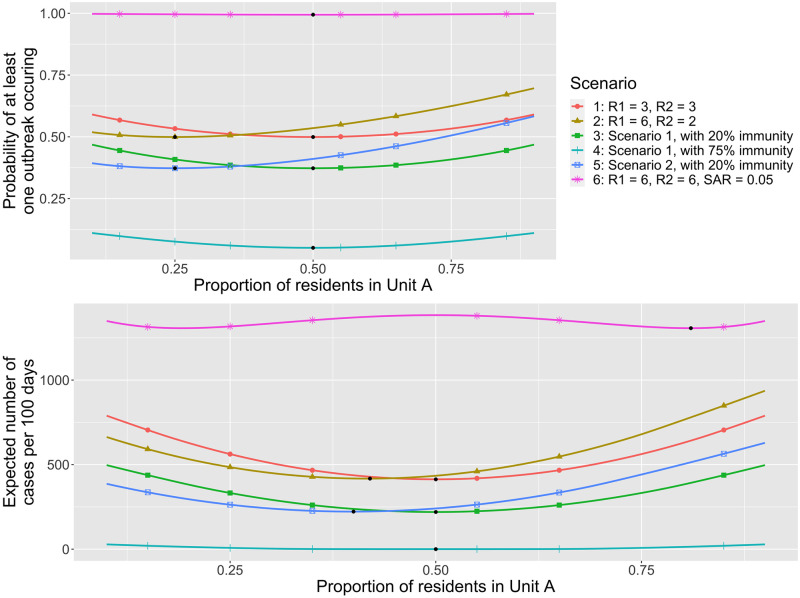
Optimal distribution of residents between two independent residential units. (Top) Probability that an outbreak occurs in at least one of two residential units within 100 days. We denote the residential units as ‘Unit A’ and ‘Unit B’. Results are shown as a proportion of the population that is housed in Unit A. Density-dependent control is assumed (i.e. Θ_*N*_ = Θ_*R*_ = 1). The total number of residents is assumed to be 2,000. Thus, if 20% of the population have immunity from control, a proportion of residents housed in Unit A of 0.25 corresponds to 400 susceptible residents in Unit A and 1,200 in Unit B. The colored lines represent six different scenarios as indicated by the legend. The black dots indicate the minimum possible probability. The reproduction values in the legend correspond to the reproduction number if the 2,000 residents are all susceptible and distributed equally in both residential units. In scenario 1 the transmission in the two residential units are identical. In scenario 2, Unit A has higher transmission than Unit B. Scenarios 3, 4 and 5, are identical to scenarios 1 or 2 except that a fraction of the residents in each residential unit have immunity from vaccination or prior infection. In scenario 6, which is designed to model a very risky housing environment, the probability that an infected staff member transmits infection to a resident (*α*_*ic*_ = 0.05) is increased five-fold compared to the other scenarios and the reproduction number for both residential units is high. (Bottom) The expected number of cases per 100 days for both residential units combined is shown as a function of the proportion of residents in Unit A. The black dots indicate the minimum possible expected number of cases. The bottom panel is otherwise analogous to the top panel. Except as noted above, the parameter values are identical to Fig 2.

The optimal distribution of residents between two separate residential units depends on the relative transmissibility in each unit, and the overall proportion of susceptible residents. As an illustrative example, we assume that both residential units have density-dependent control (i.e. Θ_*N*_ = Θ_*R*_ = 1). For this paradigm, the probability that an outbreak occurs is minimized when the residents are apportioned so that the reproduction number between the residential units is equalized (top of Fig 5). Thus if the residential units have the same transmission potential, the outbreak probability is minimized by keeping the residential unit occupancy balanced at 50% each (e.g. Scenario 1 of Fig 5). However in other situations the transmission potential may be imbalanced. For example, we consider a scenario in which the residents are evenly apportioned, but the reproduction numbers for residential units are six and two (Scenario 2 of Fig 5). In this case the outbreak probability is minimized if just 25% of residents are apportioned to the first residential unit that started with a reproduction number of six. At this occupancy, the reproduction number in both residential units becomes three (Section).

The optimal proportion of residents to house in each residential unit to minimize the expected number of cases from outbreaks does not follow a simple rule (Fig 5, bottom). For scenarios with moderate values for the probability of an outbreak occurring, the optimal proportion to house in each residential unit is closer to 50% than what is expected from analyzing the outbreak probability alone (Scenarios 2 and 5 in Fig 5). For scenarios with low values of the probability of an outbreak occurring, the expected number of cases due to outbreaks occurring can remain low for a wide range of the proportion housed in each residential unit (Scenario 4 in Fig 5). This is because the effective reproduction number can be kept below one across this range and thus outbreaks are not expected to occur. For high risk scenarios where the probability of an outbreak occurring is close to one, the best opportunity to minimize the number of cases is to reduce the number of residents in one residential unit to the point that a large outbreak can only occur in the more populous residential unit (Scenario 6 in Fig 5). Although the benefit of the optimal distribution in the high risk scenario appears marginal, it may be helpful for protecting a small subset of residents that have high risk of progressing to severe disease if they become infected. The intricacies of these relationships can be visualized by considering the outbreak dynamics in each residential unit separately (S5 Fig).

Importantly, our results depend on the relationship between the reproduction number and population size. As an extreme counterexample to the preceding results, if the reproduction number is independent of apportionment for the two residential units, it would be then best to move all residents to the unit with lower reproduction number. In addition, we have assumed the dispersion parameter is the same for both subpopulations. However, variability in the dispersion parameter can also impact optimal distribution, because the dispersion parameter affects the probability of an outbreak occurring after a single importation of infection.

#### Preemptive versus reactive control

When resources are constrained, there can be a trade-off between preemptive and reactive strategies for outbreak control. For example, there may be five equally sized residential units and only enough vaccine for 20% of the total population. As a preemptive approach, the number of susceptible residents in each residential unit could be reduced by 20%. An alternative reactive approach would be to monitor for introduction of infection and if evidence of uncontrolled transmission is detected, all residents in the affected residential unit would then be vaccinated as quickly as possible. Besides reactive vaccination, it may also be possible to implement a reactive depopulation strategy. However, it would be important that the residents who are removed from the outbreak are quarantined prior to moving to a new location. Otherwise reactive depopulation could seed outbreaks in new locations.

**Fig 6:**
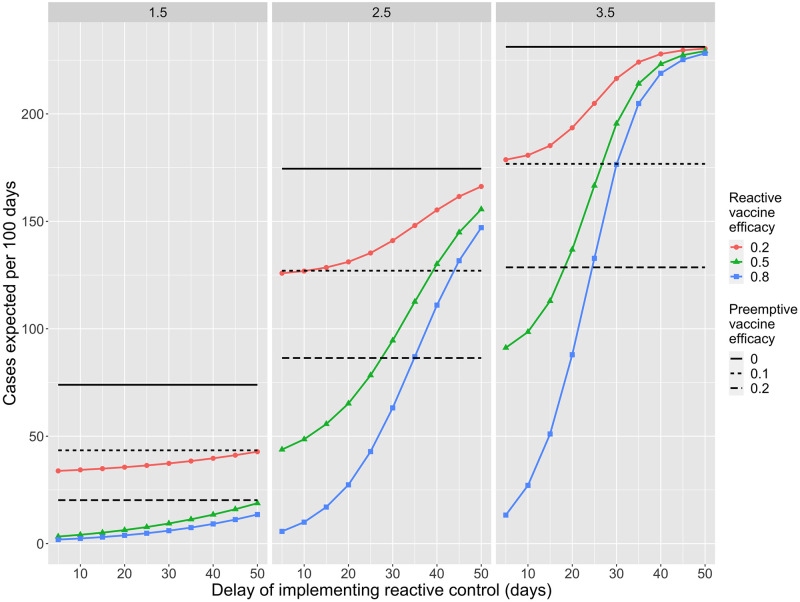
Preemptive vs reactive control. The number of cases per 100 days is shown as a function of different types of control. Each vertical panel corresponds to a different value of *R*_*B*_ as depicted on the top bar. Horizontal black bars correspond to no control being implemented. Horizontal dashed bars correspond to preemptive density-dependent control, in which immunity from density-dependent control has been achieved before an outbreak begins. The individual-level efficacy of preemptive control (*γ*) is indicated in the lower legend. Colored curves indicate the expected number of cases when reactive control is implemented once ten cases in a residential unit are identified. We model reactive control as being equally effective at reducing both disease and transmission after a predetermined control delay. The delay is depicted on the x-axis and the individual-level efficacy of reactive control (*γ*_*r*_) is depicted in the upper legend. Parameter values are otherwise identical to those in Fig 2.

When density-dependent control is assumed, the outcomes for preemptive versus reactive control are heavily dependent on the delay in achieving reactive control and the individual effectiveness of control (Fig 6). Reactive control is more likely to be superior to preemptive control if the reproduction number is low. The higher the reproduction number is, the quicker a reactive strategy will need to be deployed in order for it to have much impact. Even with a control delay of 30 days our model predicts a reactive control efficacy of 20% would be superior to preemptive control efficacy of 10% for an *R*_*B*_ of 1.5 (seen by the red line being below the dotted line in the left panel of Fig 6). However, if *R*_*B*_ were 3.5, even rapid implementation of reactive control with an efficacy of 20% would not yield better results than preemptive control with an efficacy of 10% (seen by the red line being above the dotted line in the right panel of Fig 6).

Our definition of preemptive control efficacy combines the impact of preemptive coverage and the individual efficacy for preventing transmission. For example, if two third of residents accept an infection-preventing vaccine and the vaccine decreases transmission by 75% then the preemptive control efficacy is 50%. Similarly, the reactive control efficacy is meant to incorporate a combination of effects such as vaccine hesitancy, and imperfect vaccine-induced immunity. One caveat of these results is that we have assumed that there is always enough resources available to implement reactive control. If there are outbreaks in multiple residential units that require a reactive response, then the reactive control efficacy may decrease substantially due to resource limitations. Also, implementation of reactive control may expose staff to extra health risks.

## Discussion

The burden of COVID-19 within prisons, nursing homes and other congregate settings has provided a devastating reminder of the fragility of congregate settings. To reduce unnecessary death, disease, and economic loss, policies are needed to prevent outbreaks from occurring and to mitigate outbreaks that have already started. Besides protecting the residents of congregate settings, interventions that reduce outbreak potential also reduce strain on local health systems and spillover infections in the community. [38] Outbreaks in prison settings are further complicated by the additional security, training, and contractual resources needed to hospitalize an incarcerated person. An additional consideration is the strain on staff during outbreaks due to their own health risk, longer work hours, and the emotional burden of taking care of vulnerable populations. Thus the longer term implications of reducing outbreak potential include decreasing the risk of staff burnout, post-traumatic stress disorder, and loss of institutional trust.

Our model for the impact of control on outbreak dynamics can be integrated into a variety of infection control policy decisions. For example, in the context of the COVID-19 pandemic, our study of the impact of decreasing the susceptible population could provide quantitative context to decisions about how much to prioritize decarceration of prison populations, or whether additional temporary nursing homes for COVID-19 patients are needed to ensure that existing ones are not overburdened with transmission. In particular, we found that relatively small reductions in the susceptible population of congregate settings could have a significant impact on the overall number of cases due to outbreaks. A multiplicative effect may occur due to a combination of decreasing the rate of importation of infections, the probability that an introduction leads to uncontrolled transmission and the size of any outbreaks that occur (Fig 1). Consideration of how populations can be divided into distinct residential units as well as the possibility of reactive interventions once an outbreak begins provides further opportunities for disease control.

In order to frame our analysis of control strategies in a manner that was intuitive and transparent, we made many assumptions. Depending on specific circumstances of spread in congregate settings, some assumptions will be more relevant than others. Specifically we ignore the possibility of direct transfer of infected individuals from one building to another as can inadvertently happen for individuals who are transferred during the latent period. We ignore the fluctuation of infection prevalence in the staff. Surges of infection in staff may occur due to staff-staff transmission, resident-to-staff transmission, or increases in community transmission. Similarly we ignore how staff-resident contacts may evolve over the course of a pandemic, particularly as access to personal protective equipment and education about infection control often improves with time. Our use of a SEIR transmission model assumes that within a residential unit (e.g. an isolated building) everyone is in equal contact with each other and thus ignores the finer scale structure of population dynamics. Our SEIR model also incorporates the standard assumptions of compartmental models including constant exponential rates of transitions and standard mass-action transmission. As such, our SEIR model may overly simplify the biology of transmission, such as the possibility that the relationship between duration-of-exposure and infection probability is substantially nonlinear. These assumptions ignore the possibility of seasonal effects, such as the possibility that transmission is impacted by changes in air circulation when heating or air conditioning is utilized. Except for our modification for a single reactive control intervention, The SEIR model also ignores how a large outbreak would likely inspire multiple efforts to acutely mitigate disease transmission. We also ignore the possibility that nonuniform deployment of control interventions may decrease the value of *k*. [13]

Another important caveat is that we focus solely on the disease burden of a specific congregate population during a specific time period. Thus we ignore the overall impact on disease burden in the community. On the one hand this means we do not consider how outbreaks in congregate facilities can in turn lead to outbreaks amongst staff. Staff outbreaks can then lead to increased community transmission. Thus control of outbreaks in congregate facilities may also reduce community transmission of disease. On the other hand, we do not consider that residents who transfer to the community at large due to depopulation measures can still become infected and transmit disease. Of course, the hope is that residents who transfer to the community will be more protected from acquiring disease.

Our model’s quantification of control by just a few parameters does not do justice to the complexity of public health policy decisions. Although vaccination and depopulation can be modeled similarly due to them both decreasing the size of the population susceptible to disease, the actual impact of these interventions are influenced by the different ways they impact transmission. Vaccination requires high efficacy against the circulating pathogen and uptake from the resident population. The clinical performance of vaccination and its acceptance by residents will impact the value of both the control parameter, *γ*, as well as how it impacts transmission via the control indices, Θ_*R*_ and Θ_*ρ*_. Meanwhile, depopulation may have legal, political, sociological and administrative barriers. The values of the control variables most relevant to depopulation, *γ* and Θ_*R*_, will be highly dependent on the manner in which depopulation relates to predominant mechanisms of transmission. For example, if transmission occurs due to poor ventilation then Θ_*R*_ may be small. However, if transmission is primarily due to dense living arrangements, then Θ_*R*_ may be close to one.

Quantitative application of our model to specific scenarios should include careful consideration of how breakdowns of model assumptions could impact results. For instance, the introduction of new variants of disease might lead to sudden changes in the reproduction number, and could reduce the anticipated benefit of vaccination or other control interventions. The direct applicability of our model will vary based on both the specific congregate population being considered as well as the possible range of interventions. For example, the application to nursing home populations will require consideration of whether resident-resident spread dominates, or whether a model is needed that incorporates greater staff-resident or fomite-resident transmission. In addition, the high turnover of patients in acute care facilities would require a different model structure. In prison settings our model is unlikely to be as applicable to residents in solitary cells. Instead, there is likely a core group of residents for which our model is most applicable.

It is also important to place interventions that reduce the susceptible population within a larger context of control strategies. Many scenarios may benefit from other control interventions such as improved ventilation, more frequent testing, greater adherence to social distancing, improved utilization of personal protective equipment, or cohorting of staff by residential unit. In addition, besides absolute case counts there are other important considerations for describing the impact of outbreaks on residents of congregate settings including contributions to health inequity, mental health toll, and reduction of ancillary services that are deprioritized during pandemic emergencies. Specific policy-making decisions also need to consider how control strategies will impact the ability of staff to fulfill their normal professional responsibilities, as well as protect their own health.

Our model can be adjusted to accommodate more flexible assumptions, but this would come at the cost of adding extra parameters whose values may not be readily identifiable. Importantly though, the key finding that decreasing the number of susceptible individuals in congregate settings can have a greater than linear impact on disease burden is expected to be robust to many variants of our model.

## Conclusion

Congregate settings pose a risk of large disease outbreaks. To reduce the burden that outbreaks have on residents, staff and the community at large, it is important to optimize strategies for preventing and mitigating outbreaks. We find that preemptive reduction of the size of the susceptible population via depopulation or vaccination can have a greater than linear affect on outcomes. Models can also help inform policies concerning the optimal distribution of residents, and the trade-offs between preemptive and reactive intervention strategies.

## Supporting information

S1 Fig COVID-19 incidence in California State prisons. Each panel represents one state institution. For visualization purposes, Y axes are log transformed and 7-day rolling averages of incident counts are displayed. To highlight the stochastic impact of introduction of infections, a new color is used for the incidence data whenever there is a period of no cases lasting at least 14 days. Thus the different colors approximate the consequence of individual introduction of infection into the residential community. The panels represent all institutions where at least five introductions have occurred. Names of state prisons have been removed, but are available by request. **Methodological details**: COVID-19 data for all 35 California state prisons operated by the CDCR are reported daily in a public data dashboard. Machine readable time series of these daily reports were acquired from the University of California Los Angeles COVID Behind Bars project which gathers and organizes COVID-19 data from carceral institutions across the United States. [30] Time series of incident cases were derived by taking the daily difference of reported cumulative cases. Differences in daily cumulative case counts that resulted in negative incidence estimates were ignored and incidence was estimated from the next reported cumulative case count that did not result in a negative incidence estimate.
(PNG)

S2 Fig Average time period between imported infections. Average number of days between importation of a new infection into the resident community, 1/*ϕ*, as a function of the average number of contacts a resident has with resident staff, *N*_*c*_. Colors correspond to different values for *α*_*ic*_, the probability that a resident’s contact with an infected staff member causes an infection. The prevalence of infection in the community, *P*_*com*_, is assumed to be 0.01%. The number of susceptible individuals in the congregate community, *N*_*s*_, is assumed to be 1,000.
(JPG)

S3 Fig Probability of an outbreak. The probability of an outbreak occurring as a function of the reproduction number, which is defined the average number of transmission events each new infection causes. Each panel corresponds to a different number of imported infections that may lead to an outbreak (as indicated at the top of each panel). The different colors correspond to different values of the dispersion parameter. Homogeneous transmission corresponds to *k* = ∞, and superspreading is more prevalent as *k* decreases. Plots are based on *C*_*th*_ = 10, meaning that an outbreak is defined to occur when an introduction leads to at least ten cases.
(JPG)

S4 Fig Transmission dynamics. The number of susceptible, exposed, infectious and removed residents are shown as a function of time for an outbreak in a congregate setting. The removed category includes those who have recovered from illness, those who are sick but quarantined and those who have died. The two panels represent two different values of R (as indicated at the top of each panel). A total population of 1,000 susceptible residents at the beginning of the outbreak is assumed. The average duration of each case being in the latent and infectious periods is assumed 3 and 7 days respectively (based on literature for SARS-CoV-2). The depicted outbreaks start with one exposed individual at time 0. The time step used for running the transmission dynamics model is 0.2 days, and the model is run until a negligible number of infectious individuals remain.
(JPG)

S5 Fig Outbreak dynamics depend on how residents are distributed between two residential units. The probability of an outbreak occurring in 100 days (left panels), expected number of infections for a single outbreak (middle panels), and the overall expected number of cases in 100 days (right panels) are shown as a function of the proportion of residents housed in Unit A. Top panels corresponds to Unit A and the bottom panels correspond to Unit B. Parameter values and scenarios are the same as for Fig 5.
(JPG)

## References

[1] Saloner B, Parish K, Ward JA, DiLaura G, Dolovich S. COVID-19 Cases and Deaths in Federal and State Prisons. JAMA. 2020;324(6):602–603. doi: 10.1001/jama.2020.12528 32639537 PMC7344796

[2] McMichael TM, Currie DW, Clark S, Pogosjans S, Kay M, Schwartz NG, et al. Epidemiology of Covid-19 in a Long-Term Care Facility in King County, Washington. The New England Journal of Medicine. 2020;382(21):2005–2011. doi: 10.1056/NEJMoa2005412 32220208 PMC7121761

[3] Mizumoto K, Kagaya K, Zarebski A, Chowell G. Estimating the asymptomatic proportion of coronavirus disease 2019 (COVID-19) cases on board the Diamond Princess cruise ship, Yokohama, Japan, 2020. Eurosurveillance. 2020;25(10). doi: 10.2807/1560-7917.ES.2020.25.10.2000180 32183930 PMC7078829

[4] Lu J, Gu J, Li K, Xu C, Su W, Lai Z, et al. COVID-19 Outbreak Associated with Air Conditioning in Restaurant, Guangzhou, China, 2020—Volume 26, Number 7—July 2020—Emerging Infectious Diseases journal—CDC. 10.3201/eid2607.200764 PMC7323555 32240078

[5] Park SY, Kim YM, Yi S, Lee S, Na BJ, Kim CB, et al. Coronavirus Disease Outbreak in Call Center, South Korea. Emerging Infectious Diseases. 2020;26(8):1666–1670. doi: 10.3201/eid2608.201274 32324530 PMC7392450

[6] Miller SL, Nazaroff WW, Jimenez JL, Boerstra A, Buonanno G, Dancer SJ, et al. Transmission of SARS-CoV-2 by inhalation of respiratory aerosol in the Skagit Valley Chorale superspreading event. Indoor Air. 2021;31(2):314–323. doi: 10.1111/ina.12751 32979298 PMC7537089

[7] Wodarz D, Komarova NL, Schang LM. Role of high-dose exposure in transmission hot zones as a driver of SARS-CoV-2 dynamics. Journal of the Royal Society, Interface. 2021;18(176):20200916. doi: 10.1098/rsif.2020.0916 33784886 PMC8098709

[8] Sanyaolu A, Okorie C, Marinkovic A, Patidar R, Younis K, Desai P, et al. Comorbidity and its Impact on Patients with COVID-19. SN Comprehensive Clinical Medicine. 2020;2(8):1069–1076. doi: 10.1007/s42399-020-00363-4 32838147 PMC7314621

[9] Reinhart E, Chen DL. Incarceration And Its Disseminations: COVID-19 Pandemic Lessons From Chicago’s Cook County Jail. Health Affairs (Project Hope). 2020;39(8):1412–1418. doi: 10.1377/hlthaff.2020.00652 32496864

[10] Montoya-Barthelemy AG, Lee CD, Cundiff DR, Smith EB. COVID-19 and the Correctional Environment: The American Prison as a Focal Point for Public Health. American Journal of Preventive Medicine. 2020;58(6):888–891. doi: 10.1016/j.amepre.2020.04.001 32387174 PMC7164863

[11] Henry BF. Social Distancing and Incarceration: Policy and Management Strategies to Reduce COVID-19 Transmission and Promote Health Equity Through Decarceration. Health Education & Behavior: The Official Publication of the Society for Public Health Education. 2020;47(4):536–539. doi: 10.1177/1090198120927318 32390473 PMC7347425

[12] Vest NA, Johnson OD, Nowotny KM, Brinkley-Rubinstein L. Prison population reductions and COVID-19: A latent profile analysis synthesizing recent evidence from the Texas state prison system. medRxiv. 2020; p. 2020.09.08.20190884. doi: 10.1007/s11524-020-00504-z 33337529 PMC7747775

[13] Lloyd-Smith JO, Schreiber SJ, Kopp PE, Getz WM. Superspreading and the effect of individual variation on disease emergence. Nature. 2005;438(7066):355–359. doi: 10.1038/nature04153 16292310 PMC7094981

[14] Blumberg S, Lloyd-Smith JO. Inference of R0 and Transmission Heterogeneity from the Size Distribution of Stuttering Chains. PLOS Computational Biology. 2013;9(5):e1002993. doi: 10.1371/journal.pcbi.1002993 23658504 PMC3642075

[15] Nishiura H, Yan P, Sleeman CK, Mode CJ. Estimating the transmission potential of supercritical processes based on the final size distribution of minor outbreaks. Journal of Theoretical Biology. 2012;294:48–55. doi: 10.1016/j.jtbi.2011.10.039 22079419 PMC3249525

[16] McCallum H, Barlow N, Hone J. How should pathogen transmission be modelled? Trends in Ecology & Evolution. 2001;16(6):295–300. doi: 10.1016/S0169-5347(01)02144-9 11369107

[17] Miller JC. A note on the derivation of epidemic final sizes. Bulletin of mathematical biology. 2012;74(9):2125–2141. doi: 10.1007/s11538-012-9749-6 22829179 PMC3506030

[18] Puglisi LB, Malloy GSP, Harvey TD, Brandeau ML, Wang EA. Estimation of COVID-19 basic reproduction ratio in a large urban jail in the United States. Annals of Epidemiology. 2021;53:103–105. doi: 10.1016/j.annepidem.2020.09.002 32919033 PMC7480336

[19] Mabud TS, Alves MdLD, Ko AI, Basu S, Walter KS, Cohen T, et al. Evaluating strategies for control of tuberculosis in prisons and prevention of spillover into communities: An observational and modeling study from Brazil. PLOS Medicine. 2019;16(1):e1002737. doi: 10.1371/journal.pmed.1002737 30677013 PMC6345418

[20] Lambert LA, Armstrong LR, Lobato MN, Ho C, France AM, Haddad MB. Tuberculosis in Jails and Prisons: United States, 2002–2013. American Journal of Public Health. 2016;106(12):2231–2237. doi: 10.2105/AJPH.2016.303423 27631758 PMC5104991

[21] Baussano I, Williams BG, Nunn P, Beggiato M, Fedeli U, Scano F. Tuberculosis incidence in prisons: a systematic review. PLoS medicine. 2010;7(12):e1000381. doi: 10.1371/journal.pmed.1000381 21203587 PMC3006353

[22] Lansbury LE, Brown CS, Nguyen-Van-Tam JS. Influenza in long-term care facilities. Influenza and Other Respiratory Viruses. 2017;11(5):356–366. doi: 10.1111/irv.12464 28691237 PMC5596516

[23] Rainwater-Lovett K, Chun K, Lessler J. Influenza outbreak control practices and the effectiveness of interventions in long-term care facilities: a systematic review. Influenza and Other Respiratory Viruses. 2014;8(1):74–82. doi: 10.1111/irv.12203 24373292 PMC3877675

[24] Cheng HY, Jian SW, Liu DP, Ng TC, Huang WT, Lin HH, et al. Contact Tracing Assessment of COVID-19 Transmission Dynamics in Taiwan and Risk at Different Exposure Periods Before and After Symptom Onset. JAMA internal medicine. 2020;180(9):1156–1163. doi: 10.1001/jamainternmed.2020.2020 32356867 PMC7195694

[25] He X, Lau EHY, Wu P, Deng X, Wang J, Hao X, et al. Temporal dynamics in viral shedding and transmissibility of COVID-19. Nature Medicine. 2020;26(5):672–675. doi: 10.1038/s41591-020-0869-5 32296168

[26] Lauer SA, Grantz KH, Bi Q, Jones FK, Zheng Q, Meredith HR, et al. The Incubation Period of Coronavirus Disease 2019 (COVID-19) From Publicly Reported Confirmed Cases: Estimation and Application. Annals of Internal Medicine. 2020. doi: 10.7326/M20-0504 32150748 PMC7081172

[27] Byrne AW, McEvoy D, Collins AB, Hunt K, Casey M, Barber A, et al. Inferred duration of infectious period of SARS-CoV-2: rapid scoping review and analysis of available evidence for asymptomatic and symptomatic COVID-19 cases. BMJ open. 2020;10(8):e039856. doi: 10.1136/bmjopen-2020-039856 32759252 PMC7409948

[28] Walsh KA, Spillane S, Comber L, Cardwell K, Harrington P, Connell J, et al. The duration of infectiousness of individuals infected with SARS-CoV-2. Journal of Infection. 2020;81(6):847–856. doi: 10.1016/j.jinf.2020.10.009 33049331 PMC7547320

[29] Ma Q, Liu J, Liu Q, Kang L, Liu R, Jing W, et al. Global Percentage of Asymptomatic SARS-CoV-2 Infections Among the Tested Population and Individuals With Confirmed COVID-19 Diagnosis: A Systematic Review and Meta-analysis. JAMA Network Open. 2021;4(12):e2137257. doi: 10.1001/jamanetworkopen.2021.37257 34905008 PMC8672238

[30] COVID Behind Bars Project. UCLA Law COVID-19 Behind Bars Data; 2021. Available from: https://github.com/uclalawcovid19behindbars/data.

[31] California Department of Corrections and Rehabilitation Office of Research. Population Reports; 2021. Available from: https://www.cdcr.ca.gov/research/population-reports-2/.

[32] Althouse BM, Wenger EA, Miller JC, Scarpino SV, Allard A, Hébert-Dufresne L, et al. Superspreading events in the transmission dynamics of SARS-CoV-2: Opportunities for interventions and control. PLOS Biology. 2020;18(11):e3000897. doi: 10.1371/journal.pbio.3000897 33180773 PMC7685463

[33] Adam DC, Wu P, Wong JY, Lau EHY, Tsang TK, Cauchemez S, et al. Clustering and superspreading potential of SARS-CoV-2 infections in Hong Kong. Nature Medicine. 2020;26(11):1714–1719. doi: 10.1038/s41591-020-1092-0 32943787

[34] Susswein Z, Bansal S. Characterizing superspreading of SARS-CoV-2: from mechanism to measurement. medRxiv: The Preprint Server for Health Sciences. 2020.

[35] Simpson PL, Simpson M, Adily A, Grant L, Butler T. Prison cell spatial density and infectious and communicable diseases: a systematic review. BMJ Open. 2019;9(7):e026806. doi: 10.1136/bmjopen-2018-026806 31340959 PMC6661645

[36] May RM, Anderson RM. Spatial heterogeneity and the design of immunization programs. Mathematical Biosciences. 1984;72(1):83–111. doi: 10.1016/0025-5564(84)90063-4

[37] Hethcote HW, Van Ark JW. Epidemiological models for heterogeneous populations: proportionate mixing, parameter estimation, and immunization programs. Mathematical Biosciences. 1987;84(1):85–118. doi: 10.1016/0025-5564(87)90044-7

[38] Lofgren E, Lum K, Horowitz A, Madubuonwu B, Myers K, Fefferman NH. The Epidemiological Implications of Jails for Community, Corrections Officer, and Incarcerated Population Risks from COVID-19. medRxiv. 2021; p. 2020.04.08.20058842.

