# Supplementary figures and images for: Modeling scenarios for mitigating outbreaks in congregate settings
Source: PLoS Comput Biol. 2022 Jul 20;18(7):e1010308. doi: 10.1371/journal.pcbi.1010308 (PMC9342784; doi:10.1371/journal.pcbi.1010308)

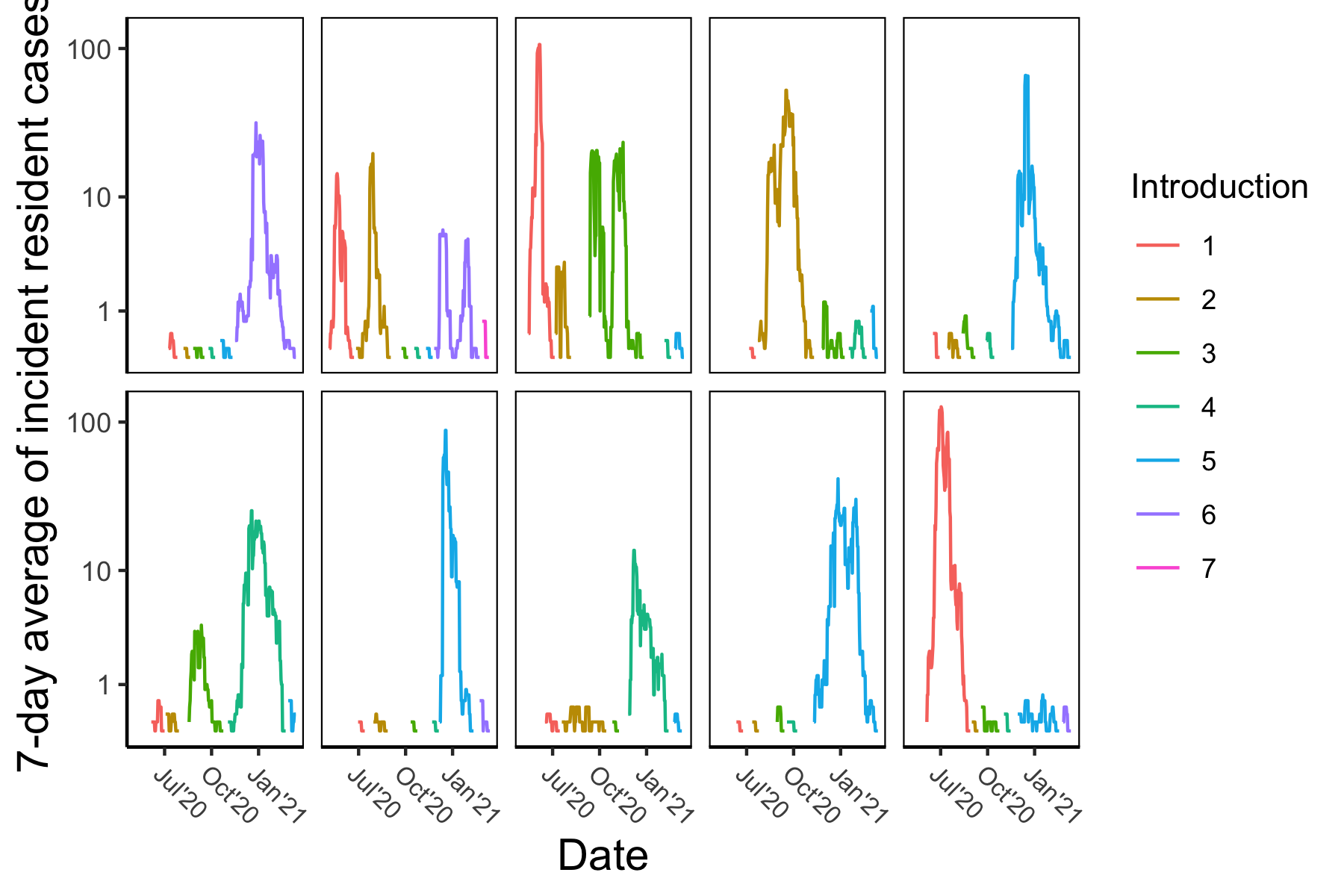

Supplement: S1 Fig — Each panel represents one state institution. For visualization purposes, Y axes are log transformed and 7-day rolling averages of incident counts are displayed. To highlight the stochastic impact of introduction of infections, a new color is used for the incidence data whenever there is a period of no cases lasting at least 14 days. Thus the different colors approximate the consequence of individual introduction of infection into the residential community. The panels represent all institutions where at least five introductions have occurred. Names of state prisons have been removed, but are available by request. Methodological details: COVID-19 data for all 35 California state prisons operated by the CDCR are reported daily in a public data dashboard. Machine readable time series of these daily reports were acquired from the University of California Los Angeles COVID Behind Bars project which gathers and organizes COVID-19 data from carceral institutions across the United States. [30] Time series of incident cases were derived by taking the daily difference of reported cumulative cases. Differences in daily cumulative case counts that resulted in negative incidence estimates were ignored and incidence was estimated from the next reported cumulative case count that did not result in a negative incidence estimate. (PNG) [file pcbi.1010308.s001.png]

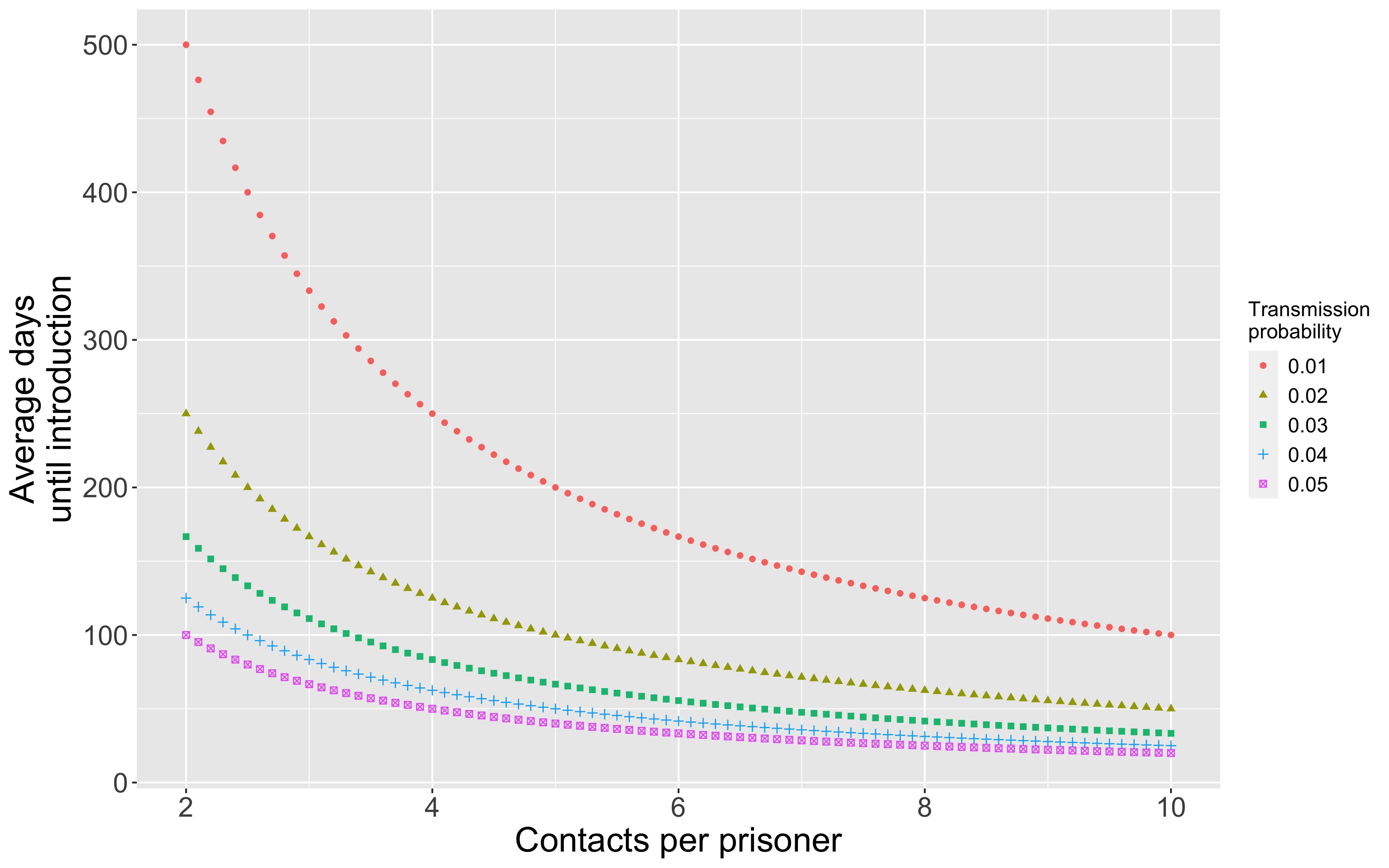

Supplement: S2 Fig — Average number of days between importation of a new infection into the resident community, 1/ϕ, as a function of the average number of contacts a resident has with resident staff, Nc. Colors correspond to different values for αic, the probability that a resident’s contact with an infected staff member causes an infection. The prevalence of infection in the community, Pcom, is assumed to be 0.01%. The number of susceptible individuals in the congregate community, Ns, is assumed to be 1,000. (JPG) [file pcbi.1010308.s002.jpg]

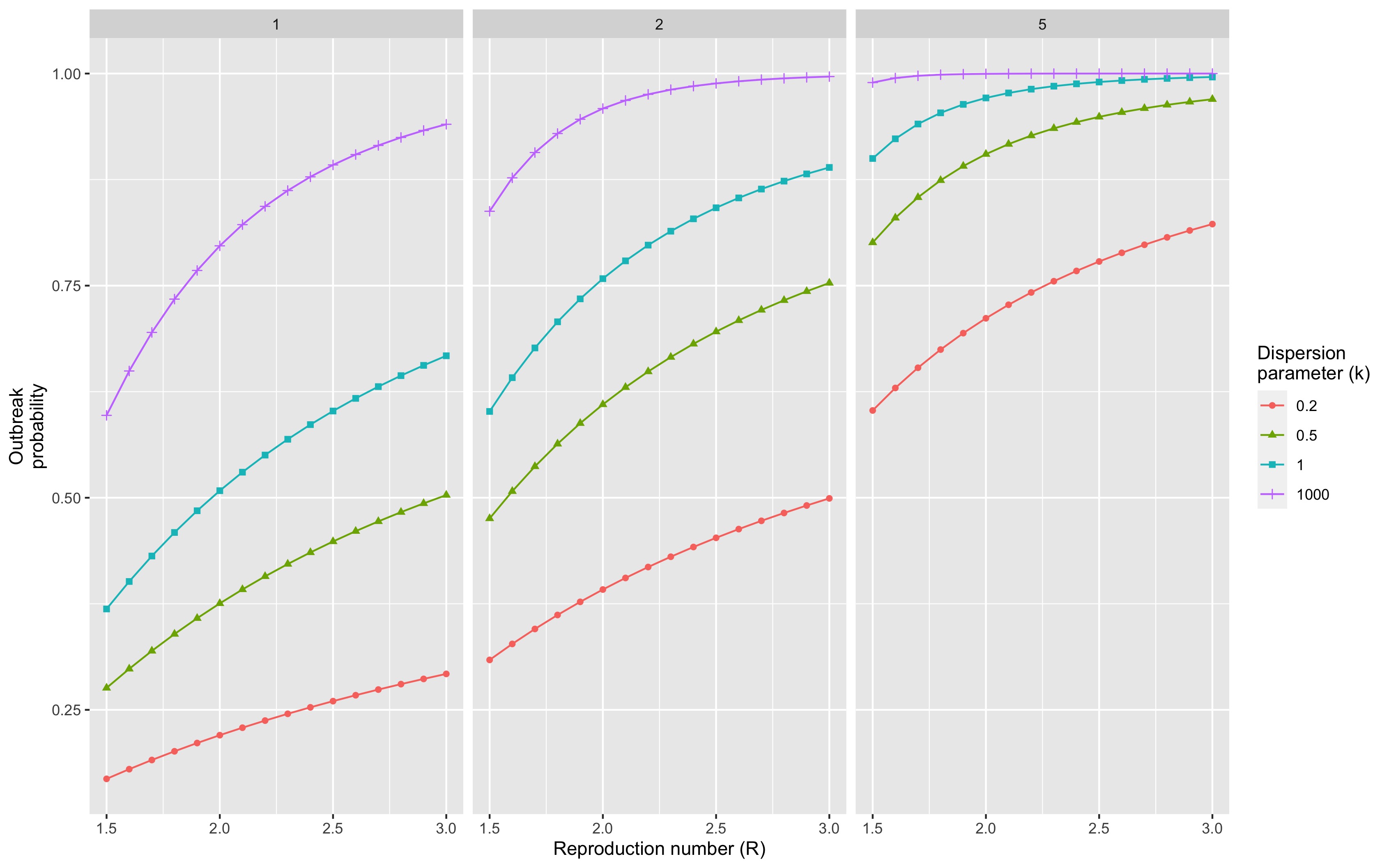

Supplement: S3 Fig — The probability of an outbreak occurring as a function of the reproduction number, which is defined the average number of transmission events each new infection causes. Each panel corresponds to a different number of imported infections that may lead to an outbreak (as indicated at the top of each panel). The different colors correspond to different values of the dispersion parameter. Homogeneous transmission corresponds to k = ∞, and superspreading is more prevalent as k decreases. Plots are based on Cth = 10, meaning that an outbreak is defined to occur when an introduction leads to at least ten cases. (JPG) [file pcbi.1010308.s003.jpg]

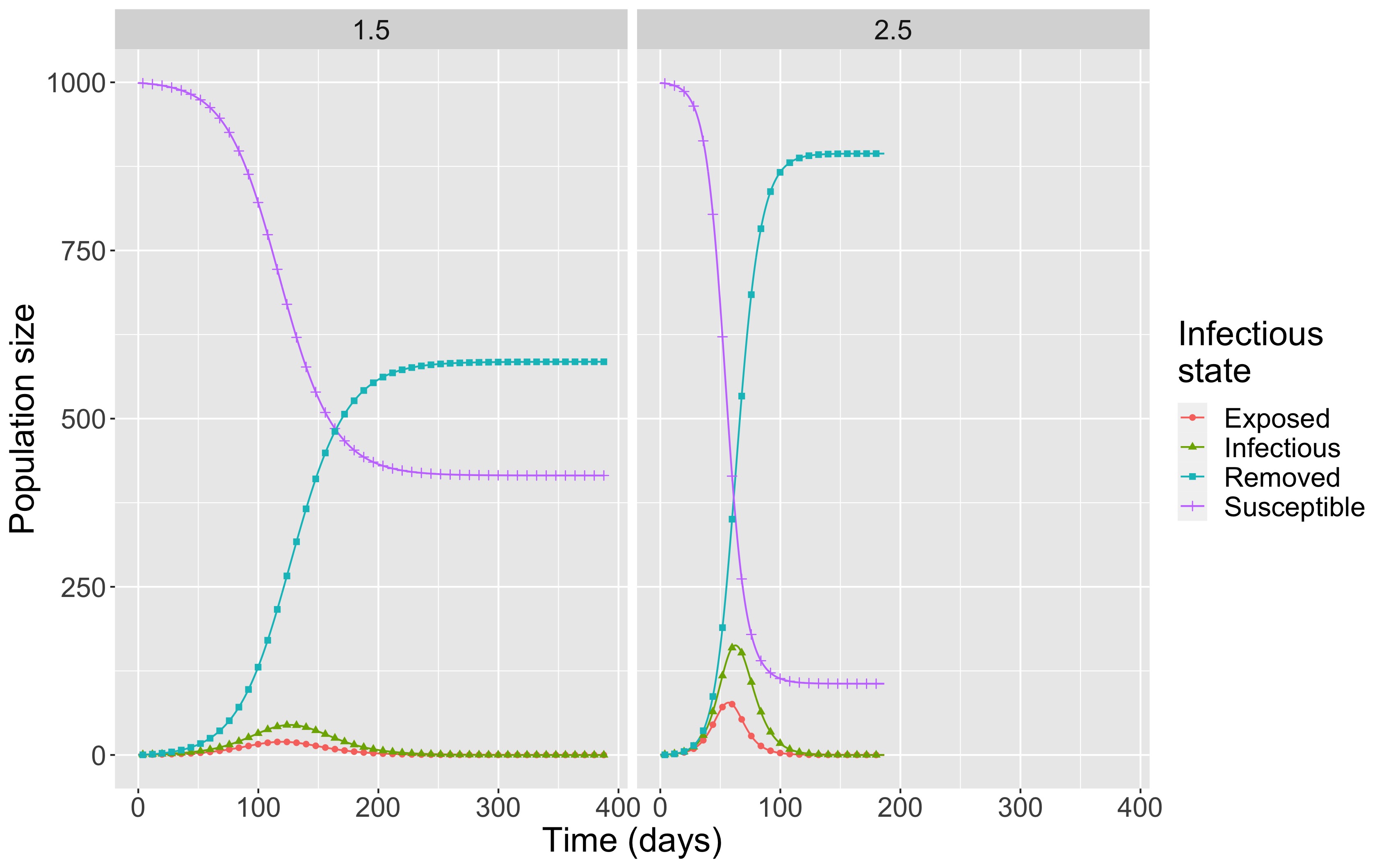

Supplement: S4 Fig — The number of susceptible, exposed, infectious and removed residents are shown as a function of time for an outbreak in a congregate setting. The removed category includes those who have recovered from illness, those who are sick but quarantined and those who have died. The two panels represent two different values of R (as indicated at the top of each panel). A total population of 1,000 susceptible residents at the beginning of the outbreak is assumed. The average duration of each case being in the latent and infectious periods is assumed 3 and 7 days respectively (based on literature for SARS-CoV-2). The depicted outbreaks start with one exposed individual at time 0. The time step used for running the transmission dynamics model is 0.2 days, and the model is run until a negligible number of infectious individuals remain. (JPG) [file pcbi.1010308.s004.jpg]

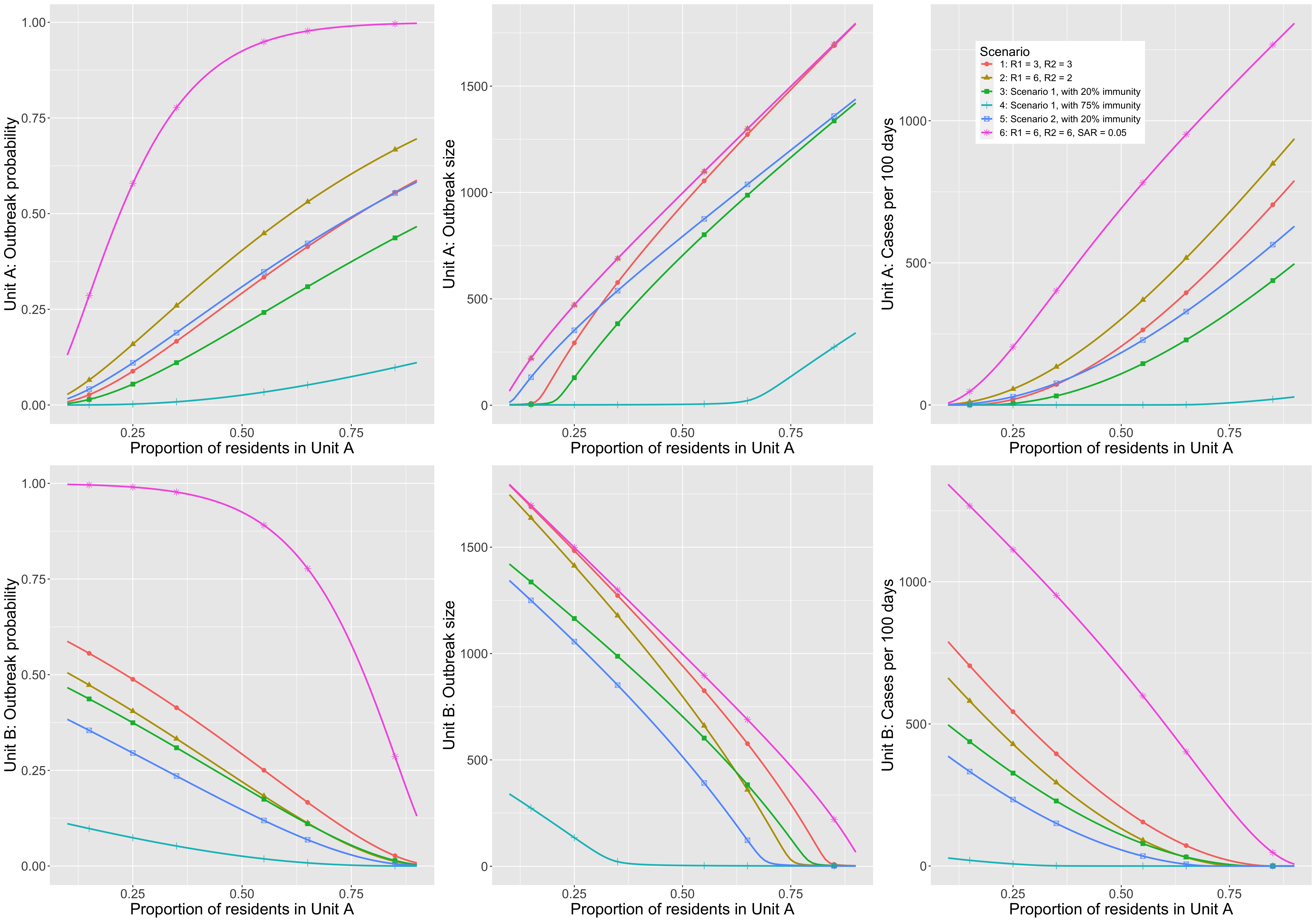

Supplement: S5 Fig — The probability of an outbreak occurring in 100 days (left panels), expected number of infections for a single outbreak (middle panels), and the overall expected number of cases in 100 days (right panels) are shown as a function of the proportion of residents housed in Unit A. Top panels corresponds to Unit A and the bottom panels correspond to Unit B. Parameter values and scenarios are the same as for Fig 5. (JPG) [file pcbi.1010308.s005.jpg]
